# Perceptions of sexual practices among the old people in Sub-Saharan African largest City, Nigeria

**DOI:** 10.1186/1742-4690-9-S1-P120

**Published:** 2012-05-25

**Authors:** Odor King, Isaac Olaseha

**Affiliations:** 1 Public Health at University of Ibadan, Abuja, Nigeria

## Introduction

Most studies on sexual behaviour in Nigeria focus on young people and adults, limited attention is paid to elderly people. Hence there is dearth of information about elderly persons’ reproductive health challenges and involvement in risky sexual activities.

## Aim

This study examined the perceptions of sexual practices among the elderly in Ibadan, Nigeria.

## Methods

The study was cross sectional in design, 400 elderly persons aged 65 years and above were selected using a three-stage sampling technique. Main outcome measures Both qualitative (FGD) and quantitative (Questionnaire) methods of data collection were used to collect relevant data on participants, sexual perception, practices and problems. The FGDs were recorded and analysed using the thematic approach, while the data from the questionnaires were analysed using descriptive and Chi-square tests.

## Results

The participants’ mean age was 71.8 (± 6.7) years. Slightly more than half, (50.5%) were males. Few (18.3%) had sex two years preceding the study. A total of 30.0% of the participants had had extramarital sex since they attained the age of 65 years. Among this subgroup, very few (7.3%) used condom. Half (50.1%) of the respondents were of the perception that condom was not meant for the elderly. Moreover, majority (68.8%) were of the perception that sex with virgin could boost immunity against STIs/HIV. Lack of interest for sexual intercourse (59.5%) was the reported main sexual problem of the respondents. Moreover, FGD participants were unanimous in their opinion that sexual dysfunction was due to ageing.

## Conclusion

Many of the elderly were involved in risky sexual practices. Therefore, health education intervention programmes such as training on safe sex practices and counselling services are needed to address the problem.

